# The impact of the COVID-19 pandemic and the changing landscape of CF on the cASPerCF trial: a real-world experience

**DOI:** 10.1186/s13063-024-08510-1

**Published:** 2024-10-02

**Authors:** Emily L. D. Chesshyre, Jacob D. Bradbury, Heather Cook, Francesca Rocchi, Kit Roes, Mark A. Turner, Roger Brüggemann, Adilia Warris

**Affiliations:** 1 MRC Centre for Medical Mycology, University of Exeter, Exeter, UK; 2 Royal Devon University Healthcare NHS Foundation Trust, Exeter, UK; 3 Clinical Trials Unit, University of Exeter, Exeter, UK; 4 Ospedale Pediatrico Bambino Gesu, Rome, Italy; 5 Radboud University Nijmegen Medical Centre, Nijmegen, the Netherlands; 6 Institute of Life Course and Medical Sciences, University of Liverpool, Liverpool, UK; 7 Centre for Women’s Health Research, Liverpool Women’s Hospital, Liverpool, UK; 8 conect4children Stichting, Utrecht, the Netherlands; 9 Great Ormond Street Hospital for Children, London, UK

Clinical outcomes and the management of children and young people with cystic fibrosis (CF) in Europe is changing significantly following the widespread roll out of highly effective CFTR modulator therapy (HEMT) such as Kaftrio (elexacaftor/tezacaftor/ivacaftor) and other combinations in people with relevant mutations. HEMT works by correcting the underlying defect in CF, the dysfunctional CF transmembrane conductance regulator (CFTR) ion transporter. Studies have shown excellent clinical efficacy with reduced pulmonary exacerbations, improved lung function and quality of life [1–4]. The altered clinical landscape gives rise to new clinical research questions such as how the phenotype of respiratory infections will change over time. In addition, alternative diagnostic methods and clinical endpoints are required with less reliance on sputum diagnostics and lung function as measured by percentage predicted forced expiratory volume in 1 s (ppFEV_1_) to define clearance of infection and beneficial effect of treatment. The potential impacts of HEMT on the design of drug trials have previously been discussed in two workshops conducted by expert working groups [5, 6] and in high profile editorials [7, 8]. Here, we provide a real-life example of the impact of HEMT. We describe the impact of the changing landscape for clinical trials involving children and young people with CF by discussing these impacts and the associated challenges the cASPerCF trial encountered. In addition, we highlight learning opportunities and provide future directions.

The cASPerCF trial was one of the non-industry proof of viability (PoV) trials under the umbrella of the connect4children (c4c) [https://conect4children.org/] project. c4c received funding from the Innovative Medicines Initiative 2 Joint Undertaking, which in turn receives support from the EU’s Horizon 2020 research and innovation programme and European Federation of Pharmaceutical Industries and Associations (EFPIA). c4c is a paediatric pan-European clinical trial network collaborating across academia, speciality networks, and the private sector to develop services that facilitate better medicines for babies, children, and young people. Its infrastructure is formed by a network of national hubs which coordinate individual sites in their countries in the delivery of clinical trials. It is overseen by a project leadership team and a project steering committee. c4c provides a strategic feasibility advice service and an education platform covering all aspects of trial design and delivery of paediatric clinical trials.

The cASPerCF trial was designed to perform a prospective validation and clinical evaluation of a new posaconazole dosing regimen for children and adolescents with CF and *Aspergillus* infection across 35 paediatric CF centres, in 10 countries in Europe. It aimed to recruit 1500 patients to be screened for the presence of *Aspergillus* infection based on a sputum culture and/or *Aspergillus*-specific IgG or IgE. Those testing positive for *Aspergillus* infection were eligible to take part in the intervention phase aimed to recruit 135 patients. The participants recruited to the intervention phase were randomised 2:1 to receive an antifungal study drug (posaconazole) or not to receive the study drug, respectively. The first site initiation visits were planned for April 2020 followed by participant recruitment starting in July 2020 (screening phase) and October 2020 (intervention phase). Unfortunately, the COVID-19 pandemic hit Europe at a critical time during study set up (February 2020) and significantly delayed the opening of the trial. For research staff, it led to a change in working patterns due to factors such as the move to fully remote working, redirection of staff and resources to COVID-19 studies, and redeployment of staff and resources to acute clinical care, factors which affected clinical research trials worldwide [9–11], with a disproportionate effect on paediatric trials [12, 13]. The first participant was recruited to the screening phase in December 2020, a delay of 6 months. In April 2021, 4 months later, a significant change in the routine clinical care of children and young people with CF became evident with the widespread roll out of Kaftrio to children over 12 years old from April 2021. The treatment with Kaftrio resulted in [1] reduced sputum production, [2] a drug-drug interaction with the study drug, and [3] improved lung function and quality of life. While this was tremendously beneficial for patients, these consequences seriously affected participant recruitment. We will discuss how these 3 consequences of the roll out of Kaftrio impacted the cASPerCF trial, the potential mitigations which were considered, and future perspectives.

The c4c project has a fixed budget and is time limited. The c4c project leadership and the cASPerCF team met each month to review progress with the trial. The c4c project has a trial commissioning committee that includes independent members to assess the viability of all the funded trials. By December 31, 2021, 29 participants had been screened (of 1500) and 1 had been recruited to the intervention phase (of 135). The c4c trial commissioning committee recognised that recruitment was not viable within the resources and time available to the project. The effects and resource implications of the suggested mitigations could not be estimated with sufficient precision to justify continued funding of the trial. Accordingly, funding from c4c was withdrawn on February 21, 2022. Alternative funding was not available, so the sponsor decided to close the study on the same date.

The reduction in the amount of sputum produced in children and young people with CF commenced on HEMT, a hugely positive clinical effect of HEMT, resulted in the difficulty of obtaining spontaneous produced sputum samples for culture to diagnose *Aspergillus* infection. Sputum culture has long been the standard method to diagnose respiratory infections in CF, including *Aspergillus* infection, and was an inclusion criterion for both the screening and intervention phase of the cASPerCF trial to detect infection (prevalence, a secondary objective of the trial) as well as assessing clearance of *Aspergillus* from the airways (a primary endpoint of the trial). The experience of the local study staff was that after patients were started on HEMT, they were less likely to be able to spontaneously produce a sputum sample. The trial was set up with performing an induced sputum procedure as the alternative if a spontaneous sputum sample could not be obtained. Although the feasibility questionnaire used to select study sites in 2019 showed that the induced sputum procedure was performed as standard of care if no spontaneous sputum sample could be obtained, the induced sputum procedure was less common practice than expected. Furthermore, the infection control implications of this aerosol generating procedure during the COVID-19 pandemic meant that patients, health care workers, and research staff were reticent to perform an induced sputum procedure. As a potential mitigation to still be able to recruit participants to the trial, the use of cough swabs was discussed with the national hubs and site representatives. Although no data is available about the sensitivity of cough swabs to detect *Aspergillus* infection, it was received as the best alternative. Subsequently, a feasibility questionnaire was sent to all participating sites in 2021 to collect evidence on the feasibility and acceptance of this alternative sample to be used instead of a sputum sample. The outcome showed that the sites were supportive of this proposal and that taking cough swabs was a feasible procedure to be performed for the cASPerCF trial.

The next key impact of Kaftrio roll out on patient recruitment was the concern about drug-drug interactions between HEMT and the study drug posaconazole. Posaconazole is a potent inhibitor of the cytochrome P450 (CYP) 3A4-mediated biotransformation comparable to itraconazole. When itraconazole is co-administered with HEMT it causes increased exposure to ivacaftor, tezacaftor, and elexacaftor. Specifically, co-administration of Kaftrio with itraconazole increased elexacaftor exposure by 2.8-fold and tezacaftor exposure by 4.0- to 4.5-fold [14]. When co-administered with itraconazole, ivacaftor exposure increased by 15.6-fold [14]. Therefore, the SmPC for Kaftrio advised that the dose of Kaftrio be reduced when co-administered with strong CYP3A inhibitors [14]. As posaconazole is a strong CYP3A inhibitor, this was anticipated during the design of the cASPerCF trial and a specific clinical management guideline on how to manage this expected drug-drug interaction was part of the cASPerCF study documents and provided to all the sites. The recommendation was to reduce the dose of Kaftrio upon initiating posaconazole treatment to prevent increased exposure of ivacaftor, tezacaftor, and elexacaftor, thereby avoiding toxicity. After the roll out of Kaftrio, potential participants were concerned about any disruption to their Kaftrio therapy and were less keen to add in a new medication which would require a substantial dose reduction of Kaftrio, albeit necessary for safety reasons and without compromising the efficacy of Kaftrio. A mitigation to take away the concern of the participants could have been to offer drug level monitoring of Kaftrio. While assessment of posaconazole levels was part of the study procedure to inform an optimal dosing regimen in children and young people with CF, therapeutic drug monitoring of Kaftrio levels was not part of the study procedure. In addition, assessment of serum concentrations of ivacaftor, tezacaftor, and elexacaftor was at that time not easily available. Of note, measuring the drug concentrations of the HEMT compounds has become available since the cASPerCF trial was terminated and will be of value to optimise the management of drug-drug interactions for future drug trials and patient care.

Lung function (ppFEV1) measured at 3, 6, and 12 months post randomisation was a secondary end point in the cASPerCF trial. Kaftrio has shown to be very effective at improving as well reducing a decline in ppFEV_1_ as early as from 14 days after starting treatment [1–4]. This confronted us with the challenge of how to be able to differentiate the benefit of posaconazole treatment on the lung function versus the beneficial effect of Kaftrio, especially if recently started. We therefore proposed to amend the eligibility criteria for the intervention phase: children and young people with CF and *Aspergillus* infection who had not yet been started on Kaftrio and not expected to be starting Kaftrio in the next 3 months or who showed a stable lung function on Kaftrio in the preceding 3 to 6 months. In addition, we considered if there were any alternative outcome measures, instead of ppFEV_1_, which could be of value. Lung clearance index (LCI) and functional MRI (fMRI) were discussed with the c4c strategic feasibility advice service as an alternative or additional clinical end point. LCI is a measure of lung physiology derived from multiple breath washout tests and is more sensitive than spirometry in identifying the presence of small airway disease [15]. It is particularly useful for monitoring disease in patients on HEMT such as shown in the PROSPECT study where no changes in spirometry were observed, but an improved LCI was observed after starting HEMT [16]. LCI has also been shown to be more sensitive than spirometry in detecting response to antibiotic treatment in children with CF [17, 18]. The procedure, which takes approximately 20 min, follows the washout of an inert tracer gas from the lungs during relaxed tidal breathing. It does not require any respiratory manoeuvres and is therefore a suitable procedure to be used in children. Functional MRI can visualise ventilation perfusion impairment without the need for contrast agents. It can detect structural abnormalities such as mucus plugging causing abnormal lung perfusion which occurs in early CF lung disease, and it has been shown to compliment LCI in detecting response to antibiotic therapy in children with CF [19, 20]. Although fMRI is non-invasive and has no radiation exposure, the length of the time needed to perform the procedure and lack of standardisation need to be considered. The premature termination of the cASPerCF trial meant that LCI and fMRI were not further considered to be included as a potential outcome measure. However, their value is likely to become more important in clinical care and as an outcome measure for future clinical trials for children and young people with CF in the era of HEMT.

Essential to the cASPerCF trial was the public and patient involvement (PPI) focus group which was involved in all aspects of the trial from the initial design of the trial through to study closure. The PPI focus group was central to the response to the key issues that the cASPerCF trial faced including the impact of the COVID-19 pandemic on people with CF, the roll out of Kaftrio, and the challenging recruitment, followed by the communication of study closure. The impact of the COVID-19 pandemic on people with CF was considerable and significantly impacted the decision to participate in the trial. People with CF went into shielding; routine appointments became remote with less frequent hospital visits and less frequent sputum and blood test sampling and monitoring. The PPI focus group identified the number of follow-up visits was a barrier to study enrolment due to a reduced outpatient visits. During the study design, children and young people used to be clinically reviewed every 3 months, and the follow-up study visits were aligned with this. But as this was changed by the COVID-19 pandemic, the study visits were no longer aligned with scheduled outpatient visits. We considered to reduce the number of follow-up visits and discussed with the c4c strategic feasibility advice service the feasibility of reducing the number of follow-up visits and/or converting to remote visits. The need for longer term safety and clinical effectiveness monitoring of posaconazole did not allow us to reduce the number of follow-up visits, although extended flexibility of the time period around the follow-up visits to take place was considered to be feasible. Another aspect identified by the PPI working group and patient and family focus groups was that after commencing HEMT, people with CF generally felt much better, with improved quality of life, meaning they were less likely to participate in a clinical trial which could potentially disrupt their daily school and home routines. We used a variety of formats such as virtual focus group discussions and questionnaires as well as engaging the wider CF community through the cASPerCF trial Twitter account (@cASPercF), the cASPerCF website (https://www.caspercf.org), and the c4c website (https://conect4children.org/studies). PPI involvement at all stages of clinical trial design and execution is vital, especially in a changing clinical landscape such as with the widespread role out of HEMT to understand new research priorities and how to adapt to these. This was clearly demonstrated in the recent survey of patients with CF and influences on clinical trial participation [21].

Ultimately, the delay in trial set up due to the COVID-19 pandemic, the similar timing with the widespread roll out of Kaftrio and subsequently poor recruitment, resulted in the decision by the c4c project leadership team to withdraw the c4c funding from the cASPerCF trial. The proposed changes to mitigate the effect of the roll out of Kaftrio could not be taken forward but will be of value in future paediatric clinical trials in CF. The challenges we encountered, such as how to diagnose respiratory infection in the absence of sputum samples and which modalities will provide the best outcome measure to assess lung function, are still to be met in the HEMT era.

The premature termination of the cASPerCF trial means that our research questions are unanswered. It remains unknown what the optimal dosing and efficacy of posaconazole is in this patient group. The question about the epidemiology of *Aspergillus* infection in children and young people with CF is likely to be changing in the HEMT era and is worth to be investigated. HEMT have been clearly shown to reduce lung disease progression, but the predisposition to respiratory infections, including *Aspergillus*, and the detection and treatment of respiratory infections remain key to the clinical management of CF [22]. The long-term outcomes of HEMT are not known, including the impact on respiratory infections. New study designs such as comparative effectiveness pragmatic trials with limited inclusion exclusion criteria, multi-centre observational studies with designs such as cross-over design, and the use of real-world data need to be considered as alternatives [23]. Trials that are affected by therapeutic revolutions may require flexible provision of resource and time for the trial.

Here, we have highlighted key challenges in the delivery of the cASPerCF trial in the era of HEMT which has resulted in an unprecedented clinical benefit for people with CF. We have identified learning outcomes and potential solutions to help inform future planning of clinical trials in children and young people with CF. We illustrate how the rapid roll out of Kaftrio to children and young people with CF across Europe, as well as the COVID-19 pandemic, led to multiple factors which impacted patient recruitment. We illustrate how we responded to the challenges including PPI engagement, multi-partner feasibility questionnaires, and use of c4c strategic feasibility advice service. Although the trial was terminated prematurely, it demonstrates how international collaboration between multiple partners (academia, regulatory agencies, health care systems, trial networks, etc.) can be done to study and improve outcomes for rare diseases in children. As a c4c PoV study, the cASPerCF trial helped to build and strengthen the infrastructure of the c4c clinical network [24], the learning from which will inform planning of future clinical trials in the c4c network. We hope that the lessons learnt from the cASPerCF trial will prove useful for future planning of paediatric clinical trials, not only in CF but in paediatric clinical trials in general.

## Data Availability

N/A.

## Abbreviations

c4c: Conect4children
CF: Cystic fibrosis
CFTR: CF transmembrane conductance regulator
CYP: Cytochrome P450
fMRI: Functional MRI
HEMT: Highly effective CFTR modulator therapy
IgG, IgE: Immunoglobulin G, immunoglobulin E
PoV: Proof of viability
LCI: Lung clearance index
PPI: Public and patient involvement
